# Interindividual Variability of Apixaban Plasma Concentrations: Influence of Clinical and Genetic Factors in a Real-Life Cohort of Atrial Fibrillation Patients

**DOI:** 10.3390/genes11040438

**Published:** 2020-04-17

**Authors:** Adela-Nicoleta Roşian, Ştefan Horia Roşian, Bela Kiss, Maria Georgia Ştefan, Adrian Pavel Trifa, Camelia Diana Ober, Ovidiu Anchidin, Anca Dana Buzoianu

**Affiliations:** 1 Department of Pharmacology, Toxicology and Clinical Pharmacology, Iuliu Haţieganu University of Medicine and Pharmacy Cluj-Napoca, 400337 Cluj-Napoca, Romania; adelarosianu@gmail.com (A.-N.R.);; 2 “Niculae Stăncioiu” Heart Institute Cluj-Napoca, 400001 Cluj-Napoca, Romania; 3 Department of Cardiology, Heart Institute, Iuliu Haţieganu University of Medicine and Pharmacy Cluj-Napoca, 400001 Cluj-Napoca, Romania; 4 Department of Toxicology, Faculty of Pharmacy, Iuliu Haţieganu University of Medicine and Pharmacy Cluj-Napoca, 400349 Cluj-Napoca, Romania; kissbela@gmail.com (B.K.); m.georgia.stefan@gmail.com (M.G.Ş.); 5 Department of Genetics, Iuliu Haţieganu University of Medicine and Pharmacy Cluj-Napoca, 400349 Cluj-Napoca, Romania; trifa.adrian@gmail.com

**Keywords:** apixaban, *ABCB_1_*, non-valvular atrial fibrillation, P-glycoprotein

## Abstract

(1) Background: Prescribing apixaban for stroke prevention has significantly increased in patients with non-valvular atrial fibrillation (NVAF). The *ABCB_1_* genotype can influence apixaban absorption and bioavailability. The aim of the present study was to assess the factors that influence apixaban’s plasma level and to establish if a certain relationship has clinical relevance. (2) Methods: Fifty-three NVAF patients were treated with 5 mg apixaban twice/day (70.0 years, range: 65–77, 60.4% men). Trough and peak plasma concentrations of apixaban were determined by liquid chromatography-tandem mass-spectrometry (LC-MS/MS), and *ABCB_1_* genotyping was performed. (3) Results: Apixaban plasma concentrations varied considerably. They were higher in women than in men (311.2 ng/dL vs. 252.2 ng/dL; *p* = 0.05) and were lower in patients with heart failure (149.4 ng/dL vs. 304.5 ng/dL; *p* < 0.01). Creatinine clearance was inversely correlated with the apixaban plasma level (Spearman correlation: *r* = −0.365; *p* = 0.007 for trough concentrations). No statistically significant differences between the genotypic groups of *ABCB_1_* rs1045642 and *ABCB_1_* rs4148738 were found in the trough or peak apixaban plasma concentrations. (4) Conclusions: Pharmacokinetic parameters are influenced by several clinical factors of which renal function is the major determinant. Plasma concentrations measured in women had higher values than those measured in men, and heart failure was associated with decreased plasma levels of apixaban.

## 1. Introduction

The risk of thromboembolic complications, especially stroke, in patients with non-valvular atrial fibrillation (NVAF), the most common type of arrhythmia, has a negative impact on quality of life and survival, implying high treatment costs [1,2,3,4]. In the past few years, prescribing apixaban for stroke and thromboembolic prevention has significantly increased in this category of patients [5].

Apixaban is a non-vitamin K oral anticoagulant (NOAC), a selective inhibitor of the Xa factor, with a rapid onset of action. Maximum plasma concentrations are achieved 4 h after oral administration and it has a half-life of 8–15 h [6].

Based on the results of two large clinical trials [7,8], the recommended dose is 5 mg twice a day, but it should be halved in patients aged over 80 years, with serum creatinine levels >1.5 mg/dL and a body weight of <60 kg [6].

Although it is currently considered to be safe and effective, apixaban also has some disadvantages related to the lack of certain parameters for routine monitoring of its effects (in patients at increased risk of bleeding or overdoses) and plasma level variations [9,10]. It is therefore mandatory to identify certain factors that will allow physicians to choose the best class and dose of anticoagulant appropriate for each patient. This goal may be achieved through the recent concept of personalized medicine based on predicting the ability of each individual to metabolize drugs.

Apixaban’s absorption is mediated by an efflux pump P-glycoprotein (P-gp) [11]. P-gp (permeability glycoprotein) is a transmembrane protein, a member of the ABC (adenosine triphosphate-binding cassette) transporter superfamily. Similar to cytochromes, there are multiple variations in P-gp function and expression, this variability being linked to a genetic component [12]. P-gp is encoded by the *ABCB_1_* gene located on chromosome 7q21 [12,13]. Several single nucleotide polymorphisms (SNPs) were identified in the promoter and exon regions of *ABCB_1_* and they were associated with the plasma concentration of P-gp substrates [14,15,16]. As a result, the hypothesis that the *ABCB_1_* genotype may influence apixaban absorption and availability is utterly plausible.

The aim of the present study was to assess the factors that influence apixaban’s plasma levels, measured at steady state, in a real-life group of patients with NVAF, treated according to guidelines recommendations. Another important goal was the periodic monitoring of adverse effects of apixaban administration in order to establish if a certain relationship has clinical relevance.

The three most common SNPs in the protein-coding region of *ABCB_1_* are rs1128503 (1236T > C, Gly412Gly), rs2032582 (2677T > G/A, Ser893Ala/Thr), and rs1045642 (3435T > C, Ile1145Ile) [17].

We chose to analyse the *ABCB_1_* rs1045642 as this SNP is the most studied polymorphism in pharmacogenetic studies on a wide range of cardiovascular drugs (atorvastatin, clopidogrel, ticagrelor, digoxin, verapamil, telmisartan) [18]. This SNP appears in 60% of European Americans and 80% of Caucasian Germans [18]. The *ABCB_1_* rs4148738 was chosen because it has been the only *ABCB_1_* polymorphism associated with dabigatran’s concentration at a genome-wide significance (*p* < 9 × 10^−8^) in a unique genome-wide association study (GWAS) on NOAC [19].

## 2. Materials and Methods

For this observational, prospective study conducted between March 2017 and June 2019, consecutive NVAF Caucasian patients, under treatment with apixaban, who attended the “Niculae Stancioiu” Emergency Heart Institute of Cluj-Napoca, were selected.

Inclusion criteria: patients of both sexes, with documented NVAF of any type (paroxysmal, persistent or permanent), who were willing to attend to the hospital for blood sampling at the specified visits and who consented to provide at least two blood samples.

Exclusion criteria: age <18 years old; failure of any dose intake one week before blood sampling; refusal of one or more blood sampling events; unwillingness to respect the visit schedule; valvular pathology (severe mitral stenosis or prosthesis); important hepatic or renal failure; serious or critical general condition; clinical situations that required treatment discontinuation (surgery, invasive procedures, overdoses, severe traumatic injury).

The anticoagulant treatment and co-prescribed medications were indicated by the attending clinician cardiologist for each patient. 

Demographic and clinical characteristics (age, sex, body weight, co-morbidities, medical treatment), a standard 12-lead ECG and echocardiographic data (GE Vivid S6 echocardiograph) were registered on individual anonymous files for each patient. The thromboembolic and bleeding risk were calculated using CHA2DS2-VASc and HAS-BLED scores, and the renal function was calculated by the Cockcroft-Gault formula. Heart failure (HF) was considered in patients who presented a history of typical signs and symptoms, underlying cardiac cause and/or ventricular dysfunction at the echocardiographic examination.

Between March and June 2017, all patients attended one visit at our clinic and provided blood samples by direct vein puncture from an antecubital vein, collected into 4 mL EDTA K3 Vacuette^®^ tubes (for through plasma concentration at 12 h from the evening apixaban dose intake and for peak plasma concentration at 2 h from the morning dose intake). The samples were immediately centrifuged at 2000× *g* for 20 min at room temperature, and plasma was frozen at −80 °C in aliquots of 0.5 mL.

A Waters Acquity liquid chromatography system coupled with a Waters TQD triple quadrupole mass spectrometer (Waters, Milford, CO, USA) was used to determine apixaban’s trough and peak plasma concentrations in the Department of Toxicology, Faculty of Pharmacy, “Iuliu Hatieganu” University of Medicine and Pharmacy. Protein precipitation was performed in a mixed methanol-water solution with hydrochloric acid. Internal standard- [13C, 2H7]-apixaban was purchased from Alsachim, Strasbourg, France. After the separation of the analyte on an Acquity BEH column, the analytes were detected by operating in positive electrospray ionization mode with multiple reaction monitoring (MRM). For data acquisition and processing MassLynx 4.2 software (Milford, MA, USA) was used.

ABCB1 genotyping was performed by the Department of Medical Genetics Laboratory of “Iuliu Hatieganu” University of Medicine and Pharmacy, Cluj-Napoca. The DNA was extracted from peripheral blood leukocytes using a commercially available kit (Wizard Genomic DNA Purification Kit, Promega, Madison, USA). *ABCB_1_* gene SNPs rs4148738 (Applied Biosystems Life Technologies assay ID C_1253813_10) and rs1045642 (custom made) were determined by real-time PCR using commercial kits, TaqMan^®^SNP Genotyping Assays, of Thermo Fisher Scientific (Waltham, MA, USA).

The entire group of patients was periodically followed-up (every six months) either through outpatient clinical visits or through a telephonic questionnaire consisting of questions about their compliance to the prescribed treatment and possible hemorrhagic or embolic complications. Major bleeding was defined according to the criteria of the International Society on Thrombosis and Haemostasis—clinically overt and associated with a decrease in hemoglobin level of ≥2.0 g/dL; need for two units of erythrocyte mass transfusion; fatal or symptomatic hemorrhages in a critical organ (intracranial, pericardial, retroperitoneal) [20]. Clinically relevant non-major (CRNM) bleeding was defined as overt bleedings that did not meet the criteria for major bleeding but were associated with medical intervention, interruption or discontinuation of apixaban, or impairment of daily life activities [21]. Minor bleeding was defined as non-clinically consequential overt bleedings that did not meet the criteria for major or CRNM bleeding.

The study was performed with the approval of the hospital’s Ethical Committee and Ethical Committee of the “Iuliu Hatieganu” University of Medicine and Pharmacy, Cluj-Napoca (registration number 106/08.03.2017) and was performed in accordance with the declaration of Helsinki. All patients signed the written informed consent form.

Statistical analysis was performed using MedCalc Statistical Software version 18.11.3 (MedCalc Software bvba, Ostend, Belgium). Data were described as the median (in the form Q1–Q3) of continuous variables that exhibited a non-normal distribution. The normality of distribution was tested using the Shapiro-Wilk test. Qualitative data were characterized by frequency and percentage. Deviations of allelic frequencies from Hardy–Weinberg equilibrium were verified using the Chi-square test. Correlations between quantitative variables were assessed using Spearman’s rank correlation coefficient. The differences between groups were compared with Mann–Whitney test, the Kruskal–Wallis test or the Chi-square test. Pearson and Spearman coefficients were used to describe the relationships between data. Multivariate analysis was carried out using linear regression (stepwise method). The dependent variables (trough plasma levels, peak plasma levels) were logarithmically transformed. We took into consideration multiple comparisons using the Bonferroni correction. A *p* value lower than 0.01 was considered statistically significant.

## 3. Results

From the patients with NVAF registered in our single tertiary emergency centre of cardiovascular diseases, we identified 94 patients treated with apixaban, for at least one month. Three of them died, 10 patients discontinued the treatment and 12 patients were administered the lower dose (2.5 mg twice-daily). Therefore, we registered 69 patients with 5 mg twice-daily, from which 53 accepted to attend the study visits and provided blood samples for the genetic analysis. Finally, the peak plasma concentration was determined in 53 patients treated with 5 mg apixaban twice-daily. Five patients refused the second blood sample, so 48 samples were used to assess the trough plasma level. 

Clinical baseline characteristics of the patients are presented in Table 1. The median age was 70.0 years, range: 65–77.

No thromboembolic or major hemorrhagic events occurred during the follow-up period (25.15 ± 0.6 months), but 11.3% of patients presented minor bleedings. At the time of sampling, patients underwent treatment for 14.67 ± 8.51 months (1–36 months).

One third of patients (39.6%) had been previously treated with vitamin-K antagonists (acenocoumarol).

The median apixaban concentration, determined by LC-MS/MS, was 132.3 (90.4; 184.2) ng/dL for trough and 287.3 (198.6; 396.8) ng/dL for peak levels. The plasma concentrations varied considerably (more than 10-fold for both); 66.03% of patients had plasma concentrations ≥ 100 ng/dL; 24.52% between 50–100 ng/dL, and 9.43% ≤ 50 ng/mL.

No significant differences in apixaban concentrations were found between patients with/without treatment with amiodarone (18.9%), beta-blockers (5.7%), propafenone (5.7%), digoxine (22.6%), statins (52.8%), antiplatelet drugs (18.9%) or nonsteroidal anti-inflammatory drugs (3.8%) (data not shown).

Plasma levels were higher in women than in men, regarding peak plasma concentration with borderline statistical significance (311.2 ng/dL (240.4; 445.1) vs. 252.2 (148.7; 333.1) ng/dL; Mann–Whitney U test: *p* = 0.05). 

Patients with heart failure had lower values of plasma concentrations, significantly for the peak levels (149.4 (133.5; 278.6) ng/dL vs. 304.5 (230.4; 438.8) ng/dL; Mann–Whitney U test: *p* < 0.01). 

None of the patients had severe renal impairment. The median glomerular filtration rate calculated according to the Cockcroft-Gault formula was 77.5 mL/min/1.72 m^2^. The creatinine clearance was inversely correlated with the apixaban plasma level, with statistical significance for trough concentrations (Spearman correlation: *r* = −0.365; *p* = 0.007).

The genotype distribution and allele frequencies of ABCB1 (in agreement with the Hardy–Weinberg equilibrium) are presented in Table 2. The 2 *ABCB_1_* SNPs were in linkage disequilibrium (D´ = 0.83 and *r*^2^ = 0.75). No statistically significant differences between the genotypic groups for any studied polymorphism, or for their haplotype combinations, were found in the trough or peak apixaban plasma concentrations. Also, statistical significance was not reached in tests for genetic models (additive, dominant or recessive). Homozygous carriers of the *ABCB_1_* rs1045642 minor allele T showed peak plasma concentrations 24% higher than those that were CC homozygous, and ABCB_1_ rs4148738 homozygous carriers of the minor allele A showed peak plasma concentrations 11% higher than those homozygous for the G allele.

A predictive model was constructed in order to find out which variables were independently linked to the apixaban plasma levels. Our model considered the age, gender, body mass index, type of AF, creatinine clearance, presence of heart failure, and rs1045642 and rs4148738 SNPs. For trough plasma levels, we obtained an *R*^2^ value of 0.407. Only the presence of heart failure and creatinine clearance were independently associated with the apixaban trough plasma levels (Table 3). For peak plasma levels, we obtained an *R*^2^ value of 0.357. Only the presence of heart failure was independently associated with the apixaban trough plasma levels (Table 4).

## 4. Discussion

From the clinician point of view, the influence of clinical, pharmacokinetic, and genetic factors on patient outcome is of significant importance. Only by integrating all these parameters can the anticoagulant treatment be managed according to each patient’s characteristics in order to avoid adverse effects: overdose and toxicity, or in contrast, the lack of a therapeutic effect. 

In this monocentric study performed on real-life NVAF patients treated with 5 mg apixaban, the plasma concentrations varied substantially (more than 10 fold). Trough plasma levels inversely correlated with creatinine clearance. Peak concentrations were higher in women and lower in patients with heart failure.

Currently, the routine monitoring of NOAC plasma concentrations is not recommended [23,24], having a well-founded dose-response relationship, although numerous studies reported plasma levels that may vary up to 20 times [25]. As in the case of the other NOACs (dabigatran, rivaroxaban, edoxaban), the therapeutic values of apixaban are not known. The producer recommends a dosage reduction to 2.5 mg in older patients with one or two dose-adjustment criteria based on extrapolation from pharmacokinetics data, not validated in “real life” patients [6]. Expected limits for 5 mg apixaban are 41–230 ng/mL for trough and 91–321 ng/mL for peak plasma concentrations [26].

The values obtained in our study using the LC-MS/MS technique are similar to those reported in the few prior individual studies that analyzed apixaban plasma levels [27,28,29,30,31]. The first studies that focused on monitoring apixaban anticoagulant activity were performed in vitro [27,28,29] or in patients with venous thromboembolism [30,31]. It was initially assumed that apixaban’s variability would be less pronounced compared to dabigatran [32,33]. In 2015, Skepholm et al. published the first study that investigated different monitoring methods in a group of real-life AF patients [34]. The results showed differences up to 10 times higher between the minimum and maximum values determined by LC-MS/MS [34]. A subsequent similar study with AF patients found a greater degree of variation in apixaban and rivaroxaban plasma concentrations (50-fold and 60-fold inter-patient variation) [35].

In the first pharmacokinetic studies performed by Leil et al. [30,31] the plasma concentration variability was associated with factors such as age, sex, race, body weight, renal function, drug interactions, and orthopedic interventions. Later, it was demonstrated that age and sex alone have a modest influence on apixaban’s pharmacokinetics and pharmacodynamics, without any clinical impact [36,37]. As in the study performed by Skeppholm et al. [34], our cohort’s age fluctuated within narrow limits and, possibly, due to this reason, no significant association between age and plasma concentration was obtained. The values measured in women were higher than those measured in men, by 18% for peak and 10% for trough levels. These data are similar to those reported in other studies published recently, which found that gender is a determining factor of a high apixaban peak level [36,38]. 

As is acknowledged, renal function is the major determinant of apixaban plasma concentrations [39,40]. The results of our study show that trough plasma levels are inversely correlated with patients’ creatinine clearance. 

A special category of patients in our cohort was represented by those with heart failure. They had lower apixaban plasma concentrations. Apixaban has low hydrosolubility [40,41], and therefore, the systemic expansion of interstitial fluid that happens in periodic temporary decompensations of heart failure cannot explain the reduced plasma values. Other possible causes could be the systemic arterial hypoperfusion status, venous congestion, and neurohormonal activation, which negatively influences the integrity and function of the key organs, in particular the gastrointestinal tract, liver, and kidneys [42].

In our study, we did not obtain a significant relationship between *ABCB_1_* genetic polymorphism and apixaban plasma levels.

From our knowledge, until now, only three individual studies evaluated the possible influence of *ABCB_1_* SNPs on apixaban’s pharmacokinetics and the results disclosed few and heterogeneous data [43,44,45]. 

The *ABCB_1_* rs4148738 SNP was analyzed in the only GWAS done so far on NOAC and has been associated with higher dabigatran peak concentrations without influencing the clinical outcome [25]. In 2016, Dimatteo et al., in a study that included 80 Caucasian patients with NVAF, reported that all carriers of the minor allele *ABCB_1_* rs4148738 had significantly higher peak plasma concentrations [43]. Later, this association was also studied in a small group of patients with AF and acute stroke by Kryukov et al., who did not detect statistically significant differences between genotypic subgroups [44]. Our homozygous carriers of *ABCB_1_* rs4148738 minor allele A had apixaban peak levels 11% higher than those homozygous for the GG genotype, but the result did not fulfill statistical significance.

The most studied *ABCB_1_* SNP—rs1045642 was first investigated by Hoffmeyer et al., and the TT genotype was related to a significant reduction in P-gp expression and higher digoxin levels [16]. Sequentially, a large number of similar studies had inconclusive and contradictory results. Similar to the other two individual studies published so far by Kryukov et al. and Ueshima et al. [44,45], in our study, *ABCB_1_* rs1045642 was not associated with apixaban plasma concentrations. Ueshima et al. found that other genetic polymorphisms such as *ABCG_2_* rs2231142 and CYP3A5*3 rs776746 together with renal function influenced apixaban pharmacokinetics in Japanese patients [45]. 

A possible explanation for the lack of association between studied *ABCB_1_* genetic polymorphism and apixaban’s pharmacokinetics could be represented by the multiple metabolic processes that influence its plasma levels or the several routes of elimination, with the involvement of different enzymes. It is possible that other genetic polymorphisms such as *CYP3A4/5*; *ABCG_2_* can influence apixaban plasma concentrations [46,47,48]. To clarify this issue, larger genomic studies must be conducted. 

No thromboembolic or major hemorrhagic events occurred during the follow-up period. Exposure–response analysis studies as well as studies assessing the impact of genetic factors on the hemorrhagic risk associated with apixaban therapy in patients with NVAF are missing. One study published in 2014 by Leil et al. evaluated apixaban efficacy in venous thromboembolism prevention after orthopedic surgery but did not find a statistically significant relationship due to the small number of cases [31]. The few studies that investigated the pharmacokinetics of apixaban in relation to the clinical characteristics of real-life patients with AF started from the assumption that the relationship between exposure and safety/efficacy shown in the case of dabigatran (the most studied NOAC) [49] can be extrapolated in the case of apixaban [34,38]. Macha et al. recently examined apixaban levels on admission associated with the severity of ischemic stroke and proved that high plasma concentrations may be protective while low levels are independent predictors of cerebral artery occlusion [50]. In our study, most patients had plasma levels above 100 ng/mL and very few (9.43%) below 50 ng/mL. Although plasma concentrations were evaluated by a single determination, the fact that thromboembolic events did not occur during the follow-up period leads us to the supposition that the levels of apixaban were maintained within therapeutic limits. Still, we cannot assert that the variability of plasma concentrations significantly influences the anticoagulant’s efficiency or the hemorrhagic risk.

The present study has several limitations: a low number of patients; a single collected sample for determining trough or peak plasma concentration; a small number of studied SNPs (which reduces the ability to analyze the influence of genetic variations on the pharmacokinetics of the studied drug); the absence of major clinical events during the follow-up period (does not allow us to obtain information regarding the influence of the pharmacokinetic impact on the patient’s clinical outcome).

## 5. Conclusions

Patients recruited from daily clinical practice showed large variations in plasma concentrations of apixaban. Pharmacokinetic parameters were influenced by several clinical factors of which renal function was the major determinant. Plasma concentrations measured in women had higher values than those measured in men, and heart failure was associated with decreased plasma levels of apixaban. *ABCB_1_* SNPs rs1045642 and rs4148738 did not significantly influence trough or peak apixaban plasma concentrations. Future studies on a larger number of patients are needed to better define the role of pharmacogenetic factors in apixaban metabolism.

## Figures and Tables

**Table 1:** Baseline characteristics of the patients included in the study.

Characteristic		Apixaban 5 mg (*n* = 53)
Median age (years)		70 (65;77)
Male *n* (%)		32 (60.4%)
BMI (kg/m^2^)		28 (24.6;31.3)
S (m^2^)		1.9 (1.6;2.1)
AF type *n* (%)	paroxysmal	11 (20.8%)
	persistent	12 (22.6%)
	permanent	30 (56.6%)
Prior VKA *n* (%)		21 (39.6%)
CHA_2_DS_2_-VAS_C_ score	1–2	10 (18.8%)
	≥3	43 (81.2%)
CrCl mL/min/1.72 m^2^		77.5 (64.2;90.0)
Minor bleeding *n* (%)		6 (11.3%)
Plasma concentrations (ng/mL)	trough	132.3 (90.4;184.2)
	peak	287.3 (198.6;396.8)

AF—atrial fibrillation; CrCl—creatinine clearance; BMI—body mass index, S—body surface; VKA—vitamin-K antagonists.

**Table 2:** Distribution of ABCB_1_ genotype and allele frequencies.

Gene			*n* (%)	MAF (%) (*n* = 53)	MAF CEU *	HWE *p*	Trough Plasma Levels Median (range)	*p* **	Peak Plasma Levels Median (range)	*p* **
ABCB_1_	rs1045642	CC	14 (26.4%)	48.11	43.4	0.98	139.4 (99.3; 187.5)	0.6	231.5 (176; 438.7)	0.8
	C > T	CT	27 (50.9%)				132.1 (87.1; 238.3)		293.5 (219.6; 370.1)	
		TT	12 (22.6%)				128 (80.1; 179.5)		305.1 (142.1; 393.2)	
	rs4148738	GG	15 (28.3%)	46.22	46.0	0.85	166.5 (99.5; 206.8)	0.5	271.4 (198.1; 442.7)	0.9
	G > A	GA	27 (50.9%)				127.4 (70.5; 194.7)		281.2 (205.6; 346.4)	
		AA	11 (20.8%)				128 (87.3; 195)		305.1 (144.7; 393.2)	

SNP single nucleotide polymorphism; MAF minor allele frequency; HWE Hardy–Weinberg equilibrium; MAF CEU *—Utah residence with northern and western European ancestry [22]; *p* **—Kruskal–Wallis test.

**Table 3:** Multivariate analysis of trough plasma levels.

	Unstandardized Coefficients B	*p*	95.0% Confidence Interval for B	
			Min	Max
(Constant)	2.783	<0.001	2.315	3.251
HF	−0.650	0.002	−1.032	−0.269
Cl Cr	−0.008	0.010	−0.014	−0.002

HF—heart failure; CrCl—creatinine clearance.

**Table 4:** Multivariate analysis of peak plasma levels.

	Unstandardized Coefficients B	*p*	95.0% Confidence Interval for B	
			Min	Max
(Constant)	2.513	<0.001	2.427	2.599
HF	−0.422	0.001	−0.650	−0.194

HF—heart failure.
